# Definition of the Clinical Characteristics of Patients with Moderate and Severe Atopic Dermatitis for Whom Narrow-Band UVB (NB-UVB) and Medium-Dose UVA1 Phototherapies Are Still Valuable Treatment Options at the Age of Biologics

**DOI:** 10.3390/jcm12093303

**Published:** 2023-05-05

**Authors:** Mariateresa Rossi, Caterina Damiani, Mariachiara Arisi, Cesare Tomasi, Francesco Tonon, Marina Venturini, Piergiacomo Calzavara-Pinton

**Affiliations:** 1 Dermatology Department, Azienda Socio Sanitaria Territoriale (ASST) Spedali Civili di Brescia, University of Brescia, 25123 Brescia, Italy; 2 Department of Experimental and Applied Medicine, Azienda Socio Sanitaria Territoriale (ASST) Spedali Civili di Brescia, University of Brescia, 25123 Brescia, Italy

**Keywords:** narrow-band UVB phototherapy, ultraviolet A1 phototherapy, dupilumab, atopic dermatitis

## Abstract

Narrow-band (NB) UVB and UVA1 have been successfully used for the treatment of atopic dermatitis (AD) since the 1980s, but the clinical indications for their use “at the age of biologics” remain to be assessed. From 2013 to 2017, 145 patients underwent a first treatment cycle with phototherapy. They achieved a median final EASI score of 9.90 with UVA1 and 13.70 with NB-UVB. The rates of patients achieving an IGA score of 0/1 persistent for at least 6 months were 33% with UVA1 and 28% with NB-UVB, and the rates with an EASI90 improvement were 10.9% with UVA1 and 11.0% with NB-UVB. The cut-off baseline EASI values for a good probability to achieve a 0/1 IGA were 24.4 with UVA1 and 24.7 with NB-UVB. A 0/1 IGA persistent for at least 6 months was more likely to be achieved by patients with a history of flares interspersed with periods of mild or no disease. From 2018, we only enrolled patients with the above-mentioned characteristics. The number of treated patients was lower, but the final EASI score, the rate of patients achieving IGA 0/1 persistent for at least 6 months, and EASI90 were significantly higher. Medium-dose UVA1 and NB-UVB phototherapies remain useful for the treatment of AD patients with a baseline EASI score lower than 24.4 and 24.7, respectively, and a medical history of flares followed by prolonged periods of complete or near-complete remission.

## 1. Introduction

Atopic dermatitis (AD) is a chronic inflammatory skin disease characterized by an impaired skin barrier, the upregulation of type 2 immune responses and increased Staphylococcus aureus colonization. Patients have dry skin, eczematous lesions and intense itching leading to excoriation and lichenification [1].

Emollients and topical drugs, i.e., corticosteroids and calcineurin inhibitors, are enough to manage patients with mild skin involvement and, until a few years ago, corticosteroids, cyclosporine, methotrexate, azathioprine and mycophenolate were the only systemic drug treatments for moderate and severe AD [1]. However, their efficacy is often poor and there are several contraindications and safety concerns, particularly if they are administered for a long time [2]. 

Phototherapies with exposures to narrow-band (312 ± 2 nm) UVB (NB-UVB) and medium-dose (30–60 J/cm^2^) UVA1 (340–400 nm) radiation have represented therapeutic alternatives to drug treatments since the 1980s. Compared to placebo or no treatment, they have been found to improve physician-rated signs and patient-reported symptoms without a difference in withdrawal due to adverse events [3]. Other main advantages include the low number of contraindications and the lack of toxicity on internal organs. However, randomized comparative controlled clinical trials (RCTs) with drug treatments have never been conducted [3], and they are not suitable for long-term and maintenance treatments because of their carcinogenic potential [4,5,6]. 

Recently, dupilumab, a fully human monoclonal antibody that binds specifically to the shared α-chain receptor subunit for interleukins (IL)-4 and IL-13, was found to be highly effective and with a good safety profile in two large RCTs [7,8]. Afterwards, other monoclonal antibodies, i.e., tralokinumab, and JAK inhibitors, i.e., baricitinib, upatacitinib and abrocitinib, were approved by the European Medicines Agency (EMA) for the treatment of AD [9]. These drugs represented a major breakthrough in the treatment paradigm for AD, which is now no longer the short-term improvement but the long-term complete or near-complete control of skin manifestations and the prevention of flares.

In this rapidly changing therapeutic landscape, the most recent guidelines and consensus papers [9,10,11] agree that phototherapies are still valuable treatment options for moderate and severe AD, but criteria for the assessment of the subgroup of AD patients who can preferentially benefit from phototherapies after the availability of new immunological treatments are not clearly established.

In 2018, which is when dupilumab became available for clinical use in Italy [8], we retrospectively reviewed the medical files of patients who underwent a first phototherapy cycle over the previous 5-year period from 2013 to 2017. We analyzed the outcome of the treatment in relation to two clinical criteria: the severity of the clinical manifestations at baseline, as measured with the EASI score; and the longitudinal medical history of the disease, by distinguishing patients with a continuous or almost-continuous course from those who present prolonged periods of absent or mild disease spontaneously or after treatments [12]. Based on the results of this first analysis (see later in Section 3), in the following 5 years (2018–2022) we only enrolled patients who had a baseline EASI score and a medical history that were associated with a good probability of remission and long-term control of the skin disease [13]. Finally, we verified the effect of these patient selection criteria by comparing the therapeutic results observed in patients treated in the period 2013–2017 with those of patients treated in the period 2018–2022. 

## 2. Materials and Methods

In 2018, we reviewed the medical files of 187 patients who underwent a first treatment cycle with NB-UVB phototherapy and medium-dose UVA1 phototherapy from 2013 to 2017 at the Photodermatology Unit of the ASST Spedali Civili University of Brescia, a tertiary referral center for AD treatment in Northern Italy. All patients had chronic atopic dermatitis for at least 3 years before screening, and topical treatment provided inadequate control or was medically inadvisable. The patients were at least 12 years of age and suffering from AD, with a baseline Eczema Area and Severity Index (EASI) of ≥7 [14,15], without a maximum value of EASI score being established. Exclusion criteria were: other inflammatory skin diseases, absolute and relative contraindications to phototherapy [16], congenital or acquired immunodeficiency syndrome and an inability to understand and sign their informed consent. Before starting phototherapy, the patients discontinued systemic drug treatments for at least 1 month and topical corticosteroids and calcineurin inhibitors for at least 2 weeks. 

At baseline, we also registered the individual longitudinal course of the disease in the previous years and dichotomized patients who reported a continuous course, i.e., skin involvement with small spontaneous variations over time and short periods of partial/good remissions after treatments, and patients who reported an intermittent course, i.e., flares interspersed with prolonged periods of absent or mild disease spontaneously or after treatments [12]. The distinction between continuous and intermittent forms is arbitrary and subject to many biases, but it was easily assessed in most cases. During the treatment cycle, oral antihistamine drugs were allowed at night if pruritus and sleep disturbance were not tolerated. The application of emollient creams was allowed as needed.

After the end of the phototherapy cycle, we also recorded the number of treatment sessions and cumulative UV dose. The patients were followed up with visits every 3 months or sooner if there was a recurrence not controlled by local therapies alone. Patients achieving an IGA of 0/1 were only allowed to use emollient creams and a limited use of topical corticosteroids, if needed. If patients had an improvement lower than EASI75 and/or an IGA score of >1, and the disease was not controlled with only topical treatments, other systemic therapies were prescribed, including new monoclonal antibodies and anti-JAK small molecules if the EASI score was ≥24 [17].

The primary outcome measures were:

- The percentage of participants achieving an Investigator’s Global Assessment (IGA) score of 0 (clear) or 1 (almost clear) [18];
- The percentage of patients achieving an IGA score of 0/1 without a relapse within 6 months from the end of treatment (EOT).

The secondary outcome measures were:

- The median final EASI score;
- The percentage of participants achieving EASI 75 (≥75% improvement from baseline EASI), EASI 50 (>50% to <75% improvement) and EASI 90 (>90% improvement);
- The percentage of patients with an EASI improvement of <50%;
- The percentage of patients with phototherapy-related adverse events;
- The percentage of patients with phototherapy-related adverse events leading to treatment discontinuation.

Afterwards, we calculated the cut-off value of the baseline EASI score that was predictive of a high probability in order to achieve a final 0/1 IGA result and the correlation of an intermittent or continuous course with a final 0/1 IGA result. Finally, we looked at the probability of obtaining a final 0/1 IGA result persistent for at least 6 months on the bases of the baseline cut-off EASI score and the history of AD course.

After 2018, we only treated patients if they had a baseline EASI score lower than the cut-off value, as calculated above (see in Section 3), and they reported an intermittent skin involvement. We recorded the personal details, individual AD characteristics, the number of treatment sessions and cumulative UV dose of these patients, and the same primary and secondary outcome measures as described above for patients treated from 2013 to 2017. 

For UVA1 exposures, we used a MediSun Xenia (Schulze & Bohm Gmbh, Bruhl, Germany) irradiation unit with UV emission strictly confined in the range from 340 to 400 nm. The radiation source of NB-UVB was a Waldmann 7001 cabinet (Waldmann Lichttechnik, Villingen-Schwenningen, Germany) equipped with 40 Philips TL-01/100W lamps (Philips, Eindhoven, Netherlands), with a peak in emission at 312 ± 2 nm. Irradiance was measured with portable broadband UV meters (Waldmann) after calibration with a Macam SR 9910 spectroradiometer (Macam Photometrics Ltd., Livingston, UK).

All patients treated with medium-dose UVA1 received a first dose of 30 J/cm^2^ and, if well tolerated, fixed daily exposures of 50 J/cm^2^ were delivered twice a week. The initial NB-UVB dose ranged between 0.1 and 0.4 J/cm^2^, according to skin phototype. The patients were treated twice-weekly on non-consecutive days, and NB-UVB doses were adjusted at each session according to the erythema response. In short, 10%, 5% or 0% increments were delivered depending on the erythema response: none, a barely perceptible or a well-defined erythema, respectively, after 48 h. With both phototherapies, treatments were continued until complete clearing was obtained, or until partial or no improvement was seen without further amelioration despite 6 additional treatments [13,16].

The database was formatted through the Microsoft Excel TM vers. 365 software and later imported from the IBM-SPSS^®^ software ver. 28.0.1 (IBM SPSS Inc., Chicago, IL, USA). The use of the Stata TM software ver. 17.0 (Stata Corporation, College Station, TX, USA) and the EpiInfo Statcalc TM software ver. 7.0 was also considered for comparisons or implementations of test output. The normality of the distributions was assessed using the Kolmogorov–Smirnov test. Categorical variables were presented as frequencies or percentages and compared with the use of the Chi-Square test and the Fisher’s exact test, as appropriate; associations of the crosstabs were verified using standardized adjusted residuals. Continuous variables were presented as means ± SD (in the case of a normal distribution), or medians and min/max (in the case of a skewed distribution), and compared with the use of a Student’s *t*-test, ANOVA, or the Mann–Whitney and Kruskal–Wallis test; correlations among variables were identified by the Pearson’s or Spearman’s rank correlation test. A two-sided α level of 0.05 was used for all tests.

The authors had full access to and take full responsibility for the integrity of the data. 

## 3. Results

The age, gender, Boston skin type, baseline EASI score and medical history (continuous/intermittent) of the AD lesions of 187 patients treated with UVA1 and NB-UVB, between 2013 and 2017, are reported in Table 1.

There were no statistically significant differences in the comparison of these features among the group of patients treated with the two phototherapies (Table 1). Both therapies were effective with a statistically significant lower median final EASI in comparison to the median baseline EASI score: final EASI score of 9.90 (0.6–50.1) with UVA1 and 13.70 (0–39.2) with NB-UVB. The measures of treatment outcome (the rate of patients achieving a 0/1 IGA result, the rate of patients with a 0/1 IGA persistent for at least 6 months, the rate of patients with EASI 90 and EASI 75 improvements) were good with both treatments (Table 1) without statistically significant differences when we compared the results obtained with medium-dose UVA1 phototherapy versus the results obtained with NB-UVB phototherapy. The median (range) number of treatment sessions was 30 (12–66) with UVA1 and 33 (16–51) with NB-UVB (*p* = NS).

**Figure 1:**
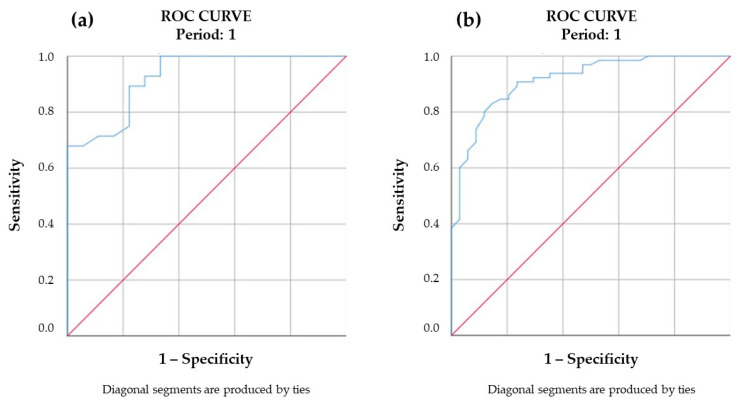
ROC curve (Receiver Operating Characteristic) of baseline EASI score (Eczema Area and Severity Index) and IGA (Investigator’s Global Assessment) 0/1 result in patients treated with medium-dose UVA1 (ultraviolet A1) phototherapy (**a**) and NB-UVB (narrow-band ultraviolet B) phototherapy (**b**). The test result variable(s): baseline EASI score has at least one tie between the positive actual state group and the negative actual state group. Statistics may be biased. The cut-off values of the baseline EASI score are 24.4 with medium-dose UVA1 phototherapy and 24.7 with NB-UVB phototherapy.

A receiver-operating characteristic (ROC) analysis of the 2013–2017 findings was performed for an exploratory evaluation of the cut-off baseline EASI score for the achievement of an IGA 0/1 treatment result, and we found that it was 24.4 (area under the curve (AUC) = 0.925 sensitivity with *p* < 0.001) with UVA1 phototherapy and 24.7 with NB-UVB phototherapy (AUC = 0.923 sensitivity with *p* < 0.001) (Figure 1).

Patients achieving an IGA 0/1 remission persistent for at least 6 months numbered 14 of 19 (73.7%); 22 of 34 (64.7%) patients had a history of intermittent disease; and 1 of 27 (3.7%) and 6 of 65 (9.2%) patients had a history of continuous disease with UVA1 phototherapy and NB-UVB phototherapy, respectively, without statistically significant differences between treatments. Afterwards, we analyzed the treatment results on the bases of both the cut-off baseline EASI scores from the ROC curves and the medical history of continuous or intermittent course; we found that the rate of patients achieving an IGA 0/1 improvement persistent for at least 6 months was statistically significantly higher for patients with a baseline EASI score lower than the cut-off scores and a history of intermittent disease (*p* < 0.001) with both phototherapies (9/13 (69.2%) patients with medium-dose UVA1 and 19/26 (73.1%) with NB-UVB), in comparison to patients with a higher baseline EASI score and/or a history of continuous moderate or severe skin involvement (Table 2).

Therefore, from 2018, we only enrolled patients with a baseline EASI lower than 24.4 with UVA1 and 24.7 with NB-UVB, and an intermittent course of skin manifestations. Therefore, the patients who had begun a first treatment cycle after 2018 were fewer than the patients who were treated for the first time in the 5 years before with both UVA1 (16 versus 46 patients) and NB-UVB phototherapy (26 versus 99 patients) (Table 1). There was no statistically significant difference in age, gender and skin phototype (Table 1). The median (range) EASI scores of patients treated with both UVA1 and NB-UVB phototherapy were significantly lower: 18.7 (8.6–27.4) versus 28.7 (8.7–52.2) (*p* < 0.001) and 18.4 (9.1–26.3) versus 30.5 (7.9–52.2) (*p* < 0.001), respectively (Table 1). There were no significant differences between the baseline EASI scores of patients treated with the two phototherapies from 2018 to 2022 (*p* = NS).

Patients treated from 2018 to 2022 underwent a significantly lower median number of treatments (30 (12–66) versus 18.5 (12–26) (*p* < 0.001) with UVA1, and 33 (16–51) versus 24 (12–30) (*p* < 0.001) with NB-UVB) and received a lower cumulative UV dose (1155.0 (360–3080) J/cm^2^ versus 770 (230–1750) J/cm^2^ with UVA1, and 16.3 (0.63–30.8) J/cm^2^ versus 11.3 (3.6–28.5) J/cm^2^ with NB-UVB) than patients treated from 2013 to 2017 with both phototherapies (Table 1). 

The rates of patients who maintained an IGA 0/1 remission for at least 6 months were higher in the group treated after 2017: 11/16 (68.7%) versus 15/46 (33%) (*p* = 0.012) with UVA1 phototherapy, and 18/26 (69.2%) and 28/99 (28%) (*p* = 0.016) with NB-UVB phototherapy. The comparisons of the rates of patients achieving a persistent IGA 0/1 result with the two phototherapies from 2018 to 2022 were never significantly different (Table 1). 

**Figure 2:**
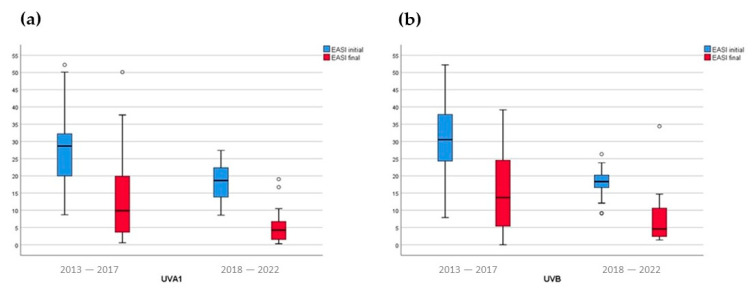
Baseline and final EASI scores (Eczema Area and Severity Index) with medium-dose UVA1 (ultraviolet A1) (**a**) and NB-UVB (narrow-band ultraviolet B) (**b**) phototherapies. The comparisons of median baseline and final EASI scores were always statistically different in all groups. The baseline EASI scores and the EASI scores of patients were significantly higher in the patients treated in the 2013–2017, in comparison to those treated in the 2018–2022 group with both phototherapies. There was no statistically significant difference comparing the baseline EASI scores and the final EASI scores of patients treated with UVA1 phototherapy or NB-UVB phototherapy in the periods 2013–2017 and 2018–2022.

The median EASI scores decreased significantly with both phototherapies from the baseline EASI score (Figure 2), but the final median EASI score was significantly lower in the patients treated between 2018 and 2022 in comparison to those treated between 2012 and 2017: 4.3 (0.3–19.0) versus 9.9 (0.6–50.1) (*p* < 0.006) with UVA1 phototherapy, and 4.6 (1.4–34.4) versus 13.7 (0–39.2) (*p* < 0.001) with NB-UVB phototherapy (Table 1). The comparison of the final EASI scores with the two phototherapies did not show differences at a statistically significant level (Table 1).

The rates of patients achieving EASI 90 were higher in the group treated from 2018 to 2022 in comparison to the group treated from 2013 to 2017 (Table 1): 5/16 (31.3%) versus 4/46 (10.9%) (*p* = 0.027) with UVA1, and 9/26 (34.6%) and 11/99 (11%) (*p* = 0.041) with NB-UVB; and the rates of patients achieving EASI 75 were 7/16 (43.8%) versus 15/46 (32.6%) (*p* = 0.02) with UVA1, and 6/26 (30.8%) and 28/99 (28.3%) (*p* = NS) with NB-UVB.

The rates of patients with at least one mild adverse effect (burns, dry skin and itching) were always low, and they were not statistically different in the patients treated in the two time intervals with both phototherapies (Table 1). They were always quickly responsive to an emollient cream. We never registered withdrawals due to adverse events, and all patients completed the treatment cycle.

## 4. Discussion

The availability of biologics and small molecules has set a new paradigm of AD treatment that is not only a complete or near-complete clearance at the end of treatment, but also a durable remission over time with the prevention of acute flares. In this new treatment landscape, the most recent guidelines still recommend NB-UVB and medium-dose UVA1 phototherapies as valuable therapeutic options, but they do not specify for which patients their use is preferable to other treatment alternatives. In the present study, the analyses of the treatment findings of patients treated between 2013 and 2017 (Table 1) showed that patients with moderate and severe disease, with baseline EASI < 24.7 with NB-UVB and <24.4 with medium-dose UVA1 (Figure 1), and a history of an intermittent course with prolonged periods of no or mild disease between acute flares, frequently achieved a complete or near-complete (IGA 0/1) remission that persisted for more than 6 months. The duration of the remission is a particularly important issue because phototherapies cannot be repeated frequently, and maintenance treatments are discouraged, due to the risk of long-term side effects, namely skin carcinogenesis. Therefore, in the present study, we found that not only the assessment of baseline cut-off scores of clinical severity [11], but also the understanding of the individual longitudinal course of AD, may improve clinical phenotyping and prognostication and facilitate a personalized therapeutic recommendation [9,12]. Indeed, it has previously been underlined that a single, static (one point in time) measurement of severity may overestimate or underestimate the true AD severity experienced by the patient, given the characteristic fluctuating severity characterized by an infinite number of combinations of disease flares, long-term persistence, and quiescence [12,19,20]. 

These patient selection criteria (a baseline EASI score lower than the above-mentioned cut-off values and a medical history characterized by intermittent disease) led to a sharp decrease in the number of patients who began a first treatment cycle, but we observed a statistically significant improvement of all parameters of treatment efficacy with higher rates of patients achieving a IGA 0/1 result and maintaining it for at least 6 months, a lower median final EASI score, and statistically higher rates of patients achieving EASI90 and EASI75 with both phototherapies.

The comparison of the efficacy measures and safety of medium-dose UVA1 and NB-UVB in the two treatment periods did not show statistically significant differences. These results are in general agreement with the findings of previous meta-analyses showing that both phototherapies are effective, without clear differences, for improving physician-rated signs and patient-reported symptoms [3,6,21].

However, we emphasize that this study was not designed as a randomized controlled comparative clinical trial and the patients were not selected for one treatment or the other, according to precise enrollment criteria [22,23]. In our daily clinical routine, the choice was made on the basis of the practical aspects of two therapies, e.g., the duration of the exposures, the heat in the irradiation unit, the lying or standing position and the cost and the duration of the waiting list at the time [24].

The results of phototherapies can be influenced by differences in the treatment protocol. We used fixed UV doses (50 J/cm^2^) for UVA1 because there is clinical evidence that medium-dose (30–60 J/cm^2^) UVA1 is more effective than low-dose regimens (10–20 J/cm^2^) and equally effective as high-dose (80–120 J/cm^2^) regimens, with a lower risk of long-term adverse effects, such as photo-aging and carcinogenic potential [6]. The NB-UVB protocol, with an initial dose based on the skin type and cautious dose increments at each exposure, is a good compromise of high efficacy and a reduced risk of adverse effects [3,9,10,25]. Moreover, the treatment choice of one phototherapy or the other cannot be driven by the present knowledge of the action mechanisms. 

Indeed, NB-UVB and UVA1 phototherapies have different photochemical and photobiological mechanisms, different penetration into the skin, intracellular targets and preferential target cell populations [26]. However, the relevant biological effects involved in the change of the pathophysiology of AD are largely similar, e.g., modulatory effects on both innate and acquired immunity with a reduction in the number and functionality of IgE-bearing intraepidermal Langerhans cells and dermal mast cells; rapid proapoptotic activity on T and B lymphocytes; a reduction in the synthesis and release of proinflammatory cytokines, such as tumor necrosis factor-alpha (TNF-α), IL-3, IL-4, IL-5, IL-12 and IL-13; and the inhibition of calcineurin phosphatase and eosinophil cationic protein (ECP), as well as antipruritic, antifibrotic, pro-pigmentary and pro-prebiotic-effects [4,26,27,28,29].

## 5. Conclusions

In this study, we found that phototherapies remain a valuable treatment option for patients with moderate and severe AD provided that they simultaneously present two clinical criteria: (1) the baseline EASI is lower than 24.4 with UVA1 and 24.7 with NB-UVB; and (2) at medical history, they report prolonged spontaneous or treatment-induced periods of complete or near-complete remission. However, their use for patients with more severe AD and/or patients with continuous involvement and quick relapses after treatments should be carefully evaluated because of the more limited efficacy, the high risk of early relapses and the risk of long-term adverse effects, namely skin carcinogenesis and skin aging, if frequent and prolonged treatment cycles are delivered. Fortunately, these patients can now benefit from new immunotherapies which are highly effective and allow long-term control of the disease [2,9]. The main limitations of this study are the retrospective, uncontrolled design and the relatively small number of patients enrolled.

## Figures and Tables

**Table 1:** Baseline main personal and clinical features and treatment results of patients who received a first treatment cycle with ultraviolet A1 (UVA1) phototherapy or narrow band-ultraviolet B (NB-UVB) phototherapy in the 2013–2017 and 2018–2022 time periods. AD = atopic dermatitis, NS = non significant.

	Patients Treated with Medium-Dose UVA1			Patients Treated with NB-UVB			Comparisons of Patients’ Groups Treated in 2013–2017 or 2018–2022 with the 2 Phototherapies	
Column	(a)	(b)	(a) vs. (b)	(c)	(d)	(c) vs. (d)	(a) vs. (c)	(b) vs. (d)
	2013–2017	2018–2022	*p*	2013–2017	2018–2022	*p*	*p*	*p*
Number	46	16		99	26			
Age (years), median (range)	26.0 (7–67)	24.5 (10–52)	NS	31 (13–73)	20.5 (14–66)	NS	NS	NS
Gender			NS			NS	NS	NS
Male (%)	12 (41.3%)	5 (31.2%)		64 (64.6%)	11 (42.3%)			
Female (%)	27 (58.7%)	11 (68.8%)		35 (35.4%)	15 (57.7%)			
Boston skin phototype, n (%)			NS			NS	NS	NS
2 (%)	12 (26.1%)	5 (31.2%)		22 (22.2%)	7 (26.9%)			
3 (%)	21 (67.4%)	10 (62.5%)		67 (67.7%)	15 (57.7%)			
4 (%)	3 (6.5%)	1 (6.3%)		10 (10.1%)	4 (15.4%)			
Medical history of AD			<0.001			<0.001	NS	NS
continuous AD, n (%)	27 (58.7%)	0 (0%)		34 (34.3%)	0 (0%)			
intermittent AD *, n (%)	19 (41.3%)	16 (100%)		65 (65.7%)	26 (100%)			
Number of treatment sessions [median (range)]	30 (12–66)	18.5 (12–26)	<0.01	33 (16–51)	24 (12–30)	<0.001	NS	NS
Cumulative UV dose (J/cm^2^) [median (range)]	1155.0 (360–3080)	770 (230–1750)	<0.01	16.3 (0.6–30.8)	11.3 (3.6–28.5)	0.02		
Patients with an IGA 0/1 result	21 (46%)	12 (75%)	0.014	42 (42%)	19 (73.1%)	0.037	NS	NS
Patients with an IGA 0/1 persistent for at least 6 months	15 (33%)	11 (69%)	0.012	28 (28%)	18 (69.2%)	0.016	NS	NS
Baseline median EASI (range)	28.7 (8.7–52.2)	18.7 (8.6–27.4)	<0.001	30.5 (7.9–52.2)	18.4 (9.1–26.3)	<0.001	NS	NS
Final median EASI (range) **	9.9 (0.6–50.1)	4.3 (0.3–19.0)	0.006	13.7 (0–39.2)	4.6 (1.4–34.4)	<0.001	NS	NS
Patients achieving EASI 90	4 (10.9%)	5 (31.3%)	0.027	11 (11%)	9 (34.6%)	0.041	NS	NS
Patients achieving EASI 75	15 (32.6%)	7 (43.8%)	0.02	28 (28.3%)	6 (30.8%)	NS	NS	NS
Patients with at least 1 adverse effect ***	8 (17.4%)	2 (12.5%)	NS	0	0	NS	NS	NS

Legend: * Intermittent AD indicates a course with flares followed by prolonged periods with no or mild disease, as it was reported by patients (POEM score <7); ** all differences of baseline versus final EASI scores were significantly different at a *p* < 0.001 level; *** Grade I burn, itching and dry skin only. Severe adverse effects or adverse effects leading to treatment discontinuation were never seen.

**Table 2:** Patients achieving an IGA score (Investigator’s Global Assessment) 0/1 improvement persistent for at least 6 months from 2013 to 2017. Treatment results were analyzed on the bases of the combination of the baseline EASI score (Eczema Area and Severity Index) and the clinical course of the skin lesions. UVA1 = ultraviolet A1; NB-UVB = narrow-band ultraviolet B.

Baseline EASI	Clinical Course	Treated Number of Patients	IGA 0/1 Persistent for at Least 6 Months	Rate (%) of Patients Achieving Persistent IGA 0/1	*p*
Patients treated with medium-dose UVA1 phototherapy (n = 46)					
<24.4	intermittent	13	9	69.2%	<0.001
<24.4	continuous	2	1	50%	
>24.5	intermittent	6	2	33.3%	
>24.5	continuous	25	3	12%	
Patients treated with NB-UVB phototherapy (n = 99)					
<24.7	intermittent	26	19	73.1%	<0.001
<24.7	continuous	6	1	16.7%	
>24.8	intermittent	8	2	25.0%	
>24.8	continuous	59	6	10.2%	

## Data Availability

The data presented in this study are available on request from the corresponding author.
